# Interrogation of the platelet-derived growth factor receptor alpha locus and corneal astigmatism in Australians of Northern European ancestry: Results of a genome-wide association study

**Published:** 2013-06-06

**Authors:** Seyhan Yazar, Aniket Mishra, Wei Ang, Lisa S. Kearns, Jenny A. Mountain, Craig Pennell, Grant W. Montgomery, Terri L. Young, Christopher J. Hammond, Stuart Macgregor, David A. Mackey, Alex W. Hewitt

**Affiliations:** 1 Centre for Ophthalmology and Visual Science, University of Western Australia, Lions Eye Institute, Perth, Australia; 2 Queensland Institute of Medical Research, Brisbane, Australia; 3 School of Women’s and Infants’ Health, University of Western Australia, Perth, Australia; 4 Centre for Eye Research Australia, University of Melbourne, Royal Victorian Eye and Ear Hospital, Melbourne, Australia; 5 Telethon Institute for Child Health Research, Centre for Child Health Research, University of Western Australia, Perth, Australia; 6 Center for Human Genetics, Duke University Medical Center, Durham, NC; 7 Duke Eye Center, Duke University, Durham, NC; 8 Department of Twin Research and Genetic Epidemiology, King’s College London, St. Thomas’ Hospital, London, UK

## Abstract

**Purpose:**

Corneal astigmatism is a common eye disorder characterized by irregularities in corneal curvature. Recently, the rs7677751 single nucleotide polymorphism (SNP) at the platelet-derived growth factor receptor alpha (*PDGFRA*) locus was found to be associated with corneal astigmatism in people of Asian ancestry. In the present study, we sought to replicate this finding and identify other genetic markers of corneal astigmatism in an Australian population of Northern European ancestry.

**Methods:**

Data from two cohorts were included in this study. The first cohort consisted of 1,013 individuals who were part of the Western Australian Pregnancy Cohort (Raine) Study: 20-year follow-up Eye Study. The second cohort comprised 1,788 individuals of 857 twin families who were recruited through the Twins Eye Study in Tasmania and the Brisbane Adolescent Twin Study. Corneal astigmatism was calculated as the absolute difference between the keratometry readings in two meridians, and genotype data were extracted from genome-wide arrays. Initially, each cohort was analyzed separately, before being combined for meta- and subsequent genome-wide pathway analysis.

**Results:**

Following meta-analysis, SNP rs7677751 at the *PDGFRA* locus had a combined p=0.32. No variant was found to be statistically significantly associated with corneal astigmatism at the genome-wide level (p<5.0×10^−8^). The SNP with strongest association was rs1164064 (p=1.86×10^−6^) on chromosome 3q13. Gene-based pathway analysis identified a significant association between the Gene Ontology “segmentation” (GO:0035282) pathway, corrected p=0.009.

**Conclusions:**

Our data suggest that the *PDGFRA* locus does not transfer a major risk of corneal astigmatism in people of Northern European ancestry. Better-powered studies are required to validate the novel putative findings of our study.

## Introduction

The majority of light refraction occurs at the air-tear film/cornea interface, as light enters the eye. Consequently, irregularities of this surface, manifesting as corneal astigmatism, can cause dramatic reductions in visual acuity. Approximately, 40% of participants from a Singapore Chinese population were astigmatic (as defined by cylindrical autorefraction readings >0.5 diopter [D]) [1,2]. In separate studies using identical definitions, more than 50% of rural Asian Indian and Persian populations were found to have astigmatism [3,4]. Interestingly, with marginally higher astigmatic groupings (either ≥0.75D or ≥1.00D), the age-adjusted prevalence of astigmatism was reported to be just over 35% in Caucasian populations from Australia and the United States [5,6].

Despite much work, the etiology of astigmatism remains poorly understood. Nonetheless, genetic and environmental factors have been suggested to have important roles in its development. Using a classical twin study, Hammond and colleagues reported that dominant and additive genetic effects accounted for approximately 46% to 79% of the phenotypic variance in corneal astigmatism [7]. Similarly, Dirani et al. found a heritability of 50% and 60% in men and women, respectively [8], while Grjibovski and colleagues calculated an overall heritability of 63% for corneal astigmatism [9].

A limited number of genome-wide association studies (GWASs) investigating corneal parameters have been conducted. Since central corneal thickness (CCT) has been found to be one of the most heritable human traits, the best studied corneal trait to date is CCT [10]. In the first published GWAS for CCT, the zinc finger 469 locus on chromosome 16q24 was identified in Australian and UK twin cohorts [11], and subsequently confirmed in other populations [12,13]. Additional quantitative trait loci for CCT have been identified on chromosomes 1p34 collagen type VIII alpha 2 gene (*COL8A2*), 6q14, 7q11, 9p23, 9q34 collagen type V alpha 1 gene (*COL5A1*), and 15q26 [12-14].

Corneal curvature (CC) is another important biometric feature, which has been recently interrogated at the genetic level. The first GWAS for keratometry (as defined as mean CC) in populations of Chinese, Malay, and Indian ancestry identified associated variants near the FK506 binding protein 12-rapamycin associated protein 1 (*FRAP1*) and platelet-derived growth factor receptor alpha (*PDGFRA*) genes [15]. Interestingly, we have recently shown that the association with CC and the *PDGFRA* locus is transferrable to people of Caucasian ancestry [16]. In a separate study, Fan and colleagues found that this locus was also associated with corneal astigmatism in a Singaporean Asian population [17]. The purpose of our present study was to investigate the role of the variants near *PDGFRA* on corneal astigmatism in people of Northern European ancestry. We also present results from a genome-wide meta-analysis for corneal astigmatism in more than 2,700 people.

## Methods

### Ethical approval

This study was conducted in accordance with the Declaration of Helsinki, and informed consent was obtained from all adult participants and at least one parent of the child participants before examination. Approval for this study was obtained from the Human Research Ethics Committees of the University of Western Australia, University of Tasmania, Royal Victorian Eye and Ear Hospital, and Queensland Institute of Medical Research.

### Sample populations

A total of 1,013 (51.3% male) unrelated individuals from the REHS and 1,788 (56.7% female) individuals of 857 twin families who were recruited through the TEST and the BATS were included in the analysis. Demographic and phenotypic characteristics of these cohorts are shown in Table 1. Two Australian cohorts of Northern European ancestry were included in this study. In both studies, corneal astigmatism was calculated as the absolute difference between horizontal and vertical keratometry readings. An inverse normal transformation was applied to the average corneal astigmatism of both eyes and used for analysis. Participants who had a pterygium or had previously undergone ocular surgery were excluded from analysis.

**Table 1:** Quality control details of genotyping in both cohorts.

Cohort	Raine	BATS/TEST	BATS/TEST
Genotyping Centre	Centre for Applied Genomics (Toronto, Ontario, Canada)	deCODE (Iceland)	CIDR (USA)
Chip	Illumina 660 K	Illumina 610 K	Illumina 610 K
# genotyped SNPs (as supplied)	657,366	592,392	589,296
mean GenCall <0.7	95,876	47,418	36,877
>5% missing	1843 (97,719)	8447 (47,950)	12,455 (37,499)
p(HWE) <10^-6^	919 (98,449)	2841 (49,616)	15,474 (51,646)
MAF<0.01 (or monomorphic)	23,370 (121,734)	33,347 (69,632)	28,607 (67,969)
# SNPs left	535,632	529,379	531,042
% genotyped SNPs	81.48%	89.36%	90.11%
Dropout rate due to QIMR SNP QC	18.52%	10.64%	9.89%

### Western Australian Pregnancy Cohort (Raine) Study: 20-year follow-up Eye Study

The first cohort comprised of participants who are enrolled in the Raine Study [18]. At the 20-year follow up, these individuals were invited to participate in the Raine Eye Health Study (REHS) and undertake a comprehensive eye examination [19]. As part of the examination, corneal curvature was measured in horizontal and vertical meridians with IOLMaster V:5 (Carl Zeiss Meditec AG, Jena, Germany). Three consecutive measurements of corneal curvature within 0.3D within each meridian were recorded with careful alignment and focus. DNA samples and consent for GWASs were available from the previous assessments. Genotype data were generated using the genome-wide Illumina 660 Quad Array at the Centre for Applied Genomics (Toronto, Ontario, Canada). As part of quality control, we investigated any individuals who were related with π >0.1875 (second- or third-degree relatives) and excluded individuals with a higher proportion of missing data. We also excluded people who had a high degree of missing genotyping data (>3%). The data were filtered for a Hardy–Weinberg equilibrium p value >5.7×10^−7^, single nucleotide polymorphism (SNP) call rate >95%, and a minor allele frequency >0.01. We conducted principal component analysis (PCA) and constructed the first five principal components for a subset of 42,888 SNPs that were not in linkage disequilibrium (LD) with each other using the EIGENSTRAT program [20]. We also performed the GWAS imputation of 22 autosomes in the MACH v1.0.16 software using the CEU samples from HapMap phase2 build 36 release 22. A linear regression model in R with a PLINK [21] interface was used to determine associations between SNPs and corneal astigmatism. The model was adjusted for age, sex, and the first two principal components that accounted for the population stratification.

### Twins Eye Study in Tasmania and Brisbane Adolescent Twin Study

The second cohort comprised participants from the Twins Eye Study in Tasmania (TEST) and the Brisbane Adolescent Twin Study (BATS) [22]. In both studies, corneal curvature was measured using a Humphrey-598 Automatic Refractor/Keratometer (Carl Zeiss Meditec, Inc., Miami, FL), and there was no significant difference between the measurements of the right and left eyes (Student *t* test, p value=0.24). In the BATS and the TEST, DNA was obtained from either saliva or peripheral blood samples. Blood was collected in tubes containing ethylenediaminetetraacetic acid and saliva samples were collected using an Oragene saliva kit (DNA Genotek, Inc., Kanata, ON, Canada). The extracted DNA from these samples was genotyped on the Illumina HumanHap 610W Quad Arrays (Illumina Inc., San Diego, CA). The majority of the BATS samples were genotyped at deCODE Genetics (Sturlugata 8; Reykjavik, Iceland) as part of a larger project. All TEST samples and a small proportion of the BATS samples (50) were genotyped at the Centre for Inherited Disease Research (CIDR; Baltimore, MD). As outlined previously, genotype data were excluded if they did not satisfy a Hardy–Weinberg equilibrium test p value ≥10^−6^, SNP call rate >95%, Illumina BeadStudio GenCall score ≥0.7, or a minor allele frequency ≥1% [23].

Ancestral outliers were corrected with PCA using the smartpca program from v3.0 of EIGENSOFT [20]. The Australian twin data were compared with all populations in HapMap phase 3 and a collection of five other GenomEUTWIN populations [24,25]. When the outliers were identified and filtered, only PC1 (the difference between the African population and others) and PC2 (the difference between the East Asian population and others) with the highest eigenvalues were used. We calculated the mean and standard deviation of the ancestral relation of the collective European population for reference PC1 and PC2 scores. Any individual who fell away from the mean by >6 times the standard deviation on PC1 and PC2 were removed. Considering the sensitivity of imputation toward missingness and SNP distribution, we conducted imputation using 469,117 common SNPs from the genotyping data present in HapMap CEU I+II data (release 22, build 36). This imputation was performed using the MACH v1.0.16b and mimimac packages [26,27], which generated association statistics for 2,543,887. These SNPs further underwent quality control with the following criteria: Hardy–Weinberg equilibrium test p value ≥10^−6^, a minor allele frequency ≥1%, and Rsq score >0.3. A total of 2,428,106 SNPs passed the filtering step and were used for further analysis. The association of these SNPs with corneal astigmatism was tested using the –fastAssoc option in MERLIN [28]. The association model was adjusted for age and sex.

In Table 2, the quality control details of the genotyping in both studies are outlined. The PCA of both population structures is shown in Appendix 1.

**Table 2:** Demographic and phenotypic characteristics of studied cohorts.

Variable	REHS	TEST/BATS
Number of subjects	1013	1788
Number of families	1013	857
Mean age in years (Range)	20	22.2 (5 to 90)
Gender (% female)	493 (48.7)	1014 (56.7)
Mean corneal astigmatism (SD; range)	0.77 (0.46; 0.08–5.16)	0.76 (0.57; 0–9)

### Joint cohort analysis

Meta-analysis of the data from two cohorts was conducted using the β-coefficients method of the METAL program [29]. Only the common SNPs imputed in both cohorts (n about 2.5 million) were included in the meta-analysis. Regional associations were generated using SNAP [30].

Pathway analysis was undertaken using Pathway-VEGAS, an extension of the recently developed gene-based analysis tool Versatile Gene-based Association Study (VEGAS) program [31]. We selected pathways from the Gene Ontology (GO) database if the pathway size ranged in 10 to 1,000 genes, which resulted in 4,628 for further analysis.

To perform pathway analysis with Pathway-VEGAS, we first conducted a gene-based test on the summary data generated from the meta-analysis. To include most regulatory effects, each gene region was defined as being 50 kb up- and downstream of a gene. VEGAS calculated the gene-based test statistics by incorporating the effects of all SNPs in the gene region by correcting the linkage disequilibrium between the SNPs through a simulation approach for the multivariate normal distribution. Since the participants in our sample are European descendents, we used the linkage disequilibrium pattern from the HapMap2 CEU reference sample. Pathway p values were calculated by summing the χ^2^ test statistics of the respective gene derived from the VEGAS p values. These summarized p values were compared with 500,000 simulations where the summarized χ^2^ test statistics of randomly drawn genes depending on the pathway size to calculate the empirical p values of the pathway. To avoid adverse effects due to clustered genes, we considered only one gene from each cluster of genes, chosen randomly, and dropped others if the distance between them was <500 kb.

## Results

**Figure 1:**
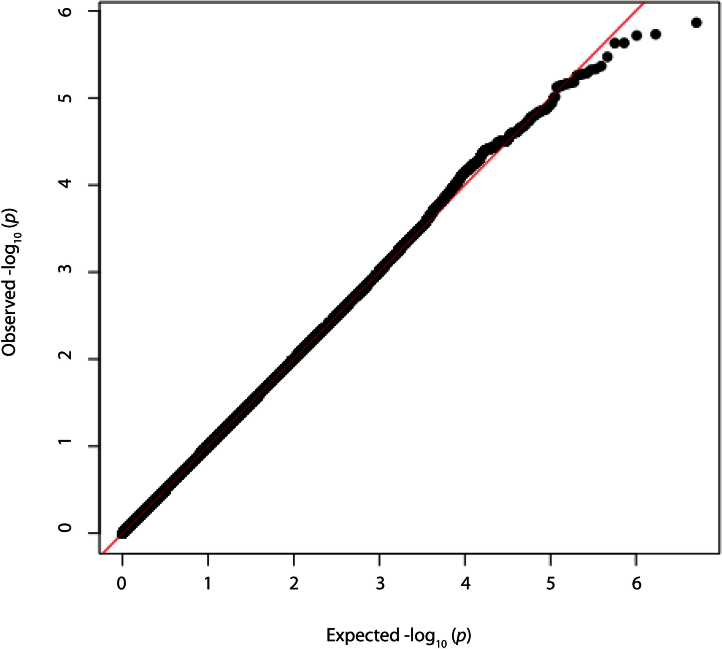
Quantile-quantile (Q-Q) plot for age and sex-adjusted genome-wide association of corneal astigmatism.

**Figure 2:**
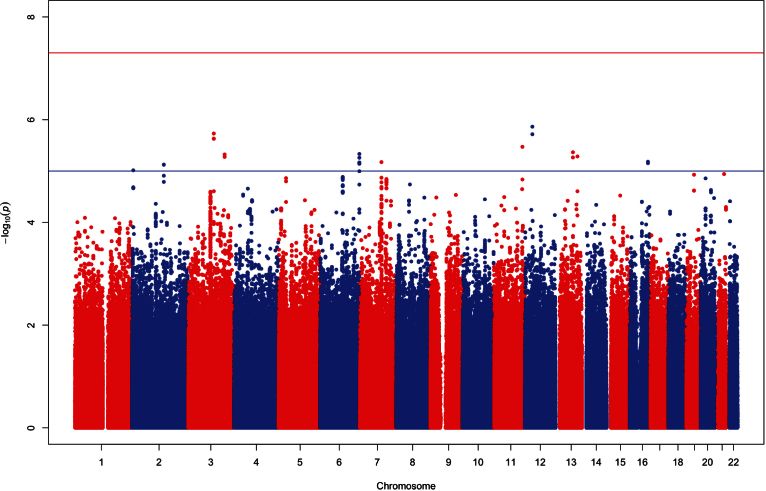
Manhattan plot of meta-analysis results. The association of single nucleotide polymorphisms and corneal astigmatism (age and sex adjusted) are plotted for each chromosome.

No loci in the TEST/BATS or Raine populations attained genome-wide significance (p<5×10^−8^). Additionally, following meta-analysis on >2.5M overlapping genotyped and imputed SNPs, no locus reached the level of genome-wide significance (Figure 1 and Figure 2). Eleven loci had a nominal threshold of suggestive significance (p<1×10^−5^). Table 3 shows details regarding the ten most significant SNPs following the meta-analysis.

**Table 3:** Strongest associated, genotyped single nucleotide polymorphisms (SNPs) for corneal astigmatism.

SNP	CHR	bp	Allele	Raine (n=1013)			TEST/BATS (n=1771)		P value	Meta-analysis		
				Effect	SEM	P value	Effect	SEM		Beta	SEM	P value
rs1151008	12	31,988,627	G	−0.138	0.046	3.07×10^−3^	−0.15	0.038	7.50×10^−5^	−0.144	0.03	1.37×10^−6^
rs1164064	3	1.11E+08	A	0.212	0.043	9.91×10^−7^	0.065	0.037	7.50×10^−2^	0.134	0.028	1.86×10^−6^
rs11841001	13	75,147,104	A	0.185	0.072	1.10×10^−2^	0.195	0.057	6.30×10^−4^	0.208	0.045	4.31×10^−6^
rs7651778	3	1.58E+08	C	0.096	0.043	2.64×10^−2^	0.139	0.036	9.30×10^−5^	0.126	0.028	4.76×10^−6^
rs11859036	16	78,863,052	A	0.145	0.043	8.74×10^−4^	0.107	0.037	3.60×10^−3^	0.128	0.029	7.03×10^−6^
rs438465	6	1.7E+08	C	−0.211	0.058	2.70×10^−4^	−0.143	0.051	5.50×10^−3^	−0.173	0.039	7.22×10^−6^
rs979976	2	1.37E+08	A	0.163	0.047	4.81×10^−4^	0.102	0.038	7.20×10^−3^	0.134	0.03	7.52×10^−6^
rs4805442	19	34,780,233	A	−0.192	0.06	1.48×10^−3^	−0.141	0.05	4.40×10^−3^	−0.168	0.038	1.18×10^−5^
rs10079889	5	33,011,247	A	0.165	0.052	1.50×10^−3^	0.124	0.043	4.30×10^−3^	0.145	0.033	1.38×10^−5^
rs2116538	2	1.37E+08	A	−0.162	0.052	1.92×10^−3^	−0.135	0.044	2.10×10^−3^	−0.146	0.034	1.63×10^−5^

Top ranking SNPs at loci identified through genome-wide meta-analysis following adjustment for age and sex as well as the results from individual cohorts.

To identify genes associated with any known pathways, we tested the VEGAS results using pathways defined in the GO database. In our analysis, the top-ranking pathways were segmentation (GO:0035282) and embryonic pattern specification (GO:0009880; Table 4). Genes involved in differentiation of mesoderm (mesogenin 1 [*MSGN1*], mesenchyme homeobox 1 [*MEOX1*], mesenchyme homeobox 2 [*MEOX2*], teratocarcinoma-derived growth factor 1 [*TDGF1*]) and anterior and posterior axis formation (homeobox D8 [*HOXD8*], homeobox A2 [*HOXA2*], homeobox B6 [*HOXB6*]) were common in both pathways.

**Table 4:** VEGAS Pathway Analysis results from gene-based meta-analysis.

GO ID	GO Term	P value*	Genes
GO:0035282	Segmentation	2.0×10^−6^ (0.009)	PSEN2, WNT3A, MSGN1, TCF7L1, ZEB2, HOXD8, TDGF1, RBPJ, LEF1, SFRP2, TBX18, DLL1, MEOX2, HOXA2, NKX3–1, SFRP1, PRKDC, MLLT3, ROR2, BMI1, EGR2, ATM, FRS2, MYF5, TBX3, PCDH8, PSEN1, MESP2, RPGRIP1L, ACD, DVL2, HES7, TCAP, KAT2A, MEOX1, HOXB6, AXIN2, MIB1, DLL3, TCF15, PAX1, POFUT1, MAFB, EP300
GO:0009880	Embryonic pattern specification	4.8×10^−5^ (0.222)	DISP1, MSGN1, TCF7L1, HOXD8, SATB2, CTNNB1, TDGF1, CXXC4, SMAD1, FGF10, SMAD5, DLL1, MEOX2, HOXA2, MLLT3, BMI1, NODAL, FRAT1, ZBTB16, FRS2, TBX3, SMAD6, RPGRIP1L, DVL2, LHX1, MEOX1, HOXB6, SMAD2, MAFB, BMP7, SIM2
GO:0007379	Segment specification	1.4×10^−4^ (0.648)	MSGN1, DLL1, MEOX2, HOXA2, MLLT3, BMI1, RPGRIP1L, DVL2, MEOX1, MAFB

Biologic pathways implicated with development of corneal astigmatism.* p values displayed first as uncorrected for number of pathways tested, with value after Bonferroni correction in parenthesis.

**Figure 3:**
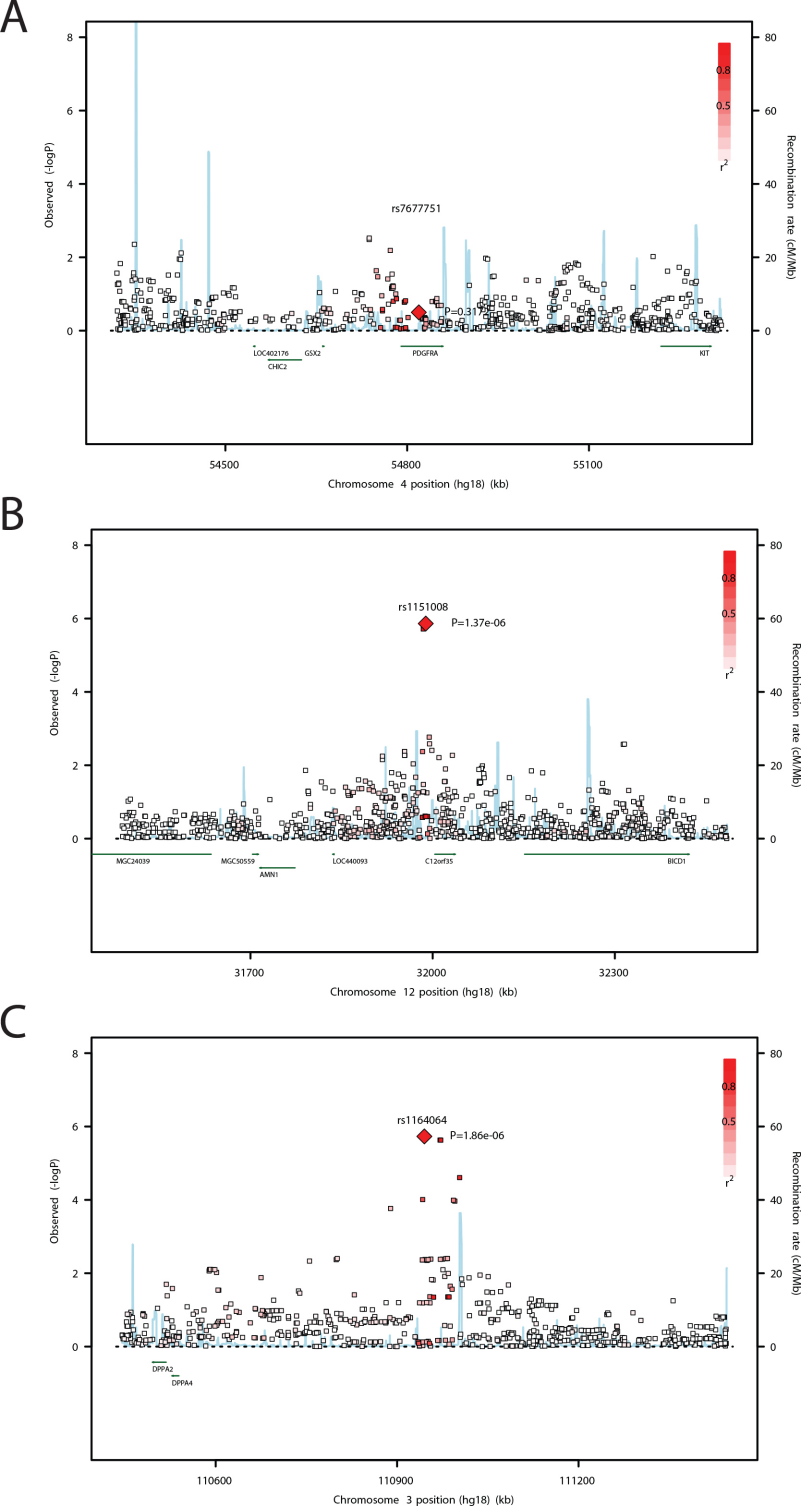
Locus-specific plots of the most significant single nucleotide polymorphisms (SNPs) in this study. These plots display the most significant result in the meta-analysis genome-wide association study (GWAS). The locus identified by Fan et al. Chromosome 4q12 (**A**) and two loci identified in this study Chromosome 12p11 (**B**) and 3q13 (**C**) are shown. SNPs are plotted as the –log10 of the p value.

We found no evidence for replication of the *PDGFRA* locus (Figure 3). In our cohorts, the previously reported top SNP in this region (rs7677751) was not significantly associated with corneal astigmatism (beta=–0.0423, standard deviation error=0.0423; p=0.32). The minor allele frequency of rs7677751 was 0.133 and 0.123 in the Raine study and the TEST/BATS, respectively. The top SNP at this locus was rs6821576 (p=0.003).

## Discussion

Understanding the molecular mechanism of cornea-related disease is useful for developing novel corrective or therapeutic strategies. Recently, through meta-analysis of five Singaporean cohorts, Fan and colleagues reported a statistically significant association between a variant (rs7677751) at the *PDGFRA* locus on chromosome 4q12 and corneal astigmatism [17]. In our present study, we found no strong evidence for transfer of risk for corneal astigmatism of this locus in two Australian cohorts of Northern European ancestry. Our results suggest there are underlying genetic differences between populations, which may account for differing development and prevalence of corneal astigmatism.

Although no SNP in our study was significantly associated with corneal astigmatism at the genome-wide level, we identified several putative loci, which reached a suggestive level of significance (p<1×10^−5^). Our strongest signal on meta-analysis (rs1151008) was located on chromosome 12p11. This SNP is approximately 100 kb centromeric to the antagonist of the mitotic exit network 1 homolog (*AMN1*) gene. *AMN1* has been shown to be important in resetting the cell cycle [32], and the *AMN1* domain of the *lysine-specific demethylase 2A* gene appears to inhibit keratinocyte growth in vitro [33].

Our second strongest-association (rs1164064) was on chromosome 3q13, near the developmental pluripotency-associated 4 (*DPPA4*) and *DPPA2* genes (Figure 3). These genes have important roles in stem cell generation and are rapidly downregulated during cellular or fetal differentiation [34]. Given that the cornea is of ectodermal origin, *DPPA4* regulates differentiation of embryonic stem cells into a primitive ectoderm lineage [35].

Following gene-based pathway analysis, we found that genes involved with segmentation and embryonic pattern specification were associated with the development of corneal astigmatism. In vertebrates, the periocular mesenchymal cells migrate into the cornea giving rise to cornea stroma during embryogenesis [36]. Interestingly, the *MSGN1*, *MEOX1*, *MEOX2*, and *TDGF1* genes identified in our pathway analysis are involved in differentiation of mesoderm. Additionally, some of the genes in these pathways are part of the *HOX* family, which included the *HOX8.1* gene that was demonstrated to be expressed during murine ocular development [37].

It is somewhat surprising that, despite our reasonable power, we were unable to replicate the association of corneal astigmatism and the rs7677751 variant [17]. We also failed to identify any locus associated with this trait at the genome-wide significance level. Our results suggest that in dissecting the genetic architecture of corneal astigmatism in people of Northern European ancestry, no major single locus will predominant, similar to other complex quantitative traits [38]. Clearly, larger, better-powered cohorts are required to intimately dissect the genetic etiology of this biometric trait.

In summary, we found no strong evidence for replication or transferability of the previously reported association between the rs7677751 variant, at the *PDGFRA* locus, and corneal astigmatism in our Australian cohorts of Northern European ancestry. We identified several putative loci, which clearly require replication in ongoing genetic or functional studies.

## Appendix 1.

Principal component analysis (PCA) plots of REHS (A) and TEST/BATS (B) population structures. To access the data, click or select the words “Appendix 1.”
